# Isolation and Characterization of *Pseudomonas aeruginosa* XR2-39 Against *Meloidogyne incognita* and Its Enhancement of Tomato Growth

**DOI:** 10.3390/microorganisms14010005

**Published:** 2025-12-19

**Authors:** Mengyu Yuan, Wuping Li, Linjuan Fan, Fan Zhang, Caiyun Wu, Xueliang Xu, Yingjuan Yao, Zhihui Zhu, Shaoqin Li, Jian Yao

**Affiliations:** 1 Hubei Insect Resources Utilization and Sustainable Pest Management Key Laboratory, College of Plant Sciences and Technology, Huazhong Agricultural University, Wuhan 430070, China; yuanmengyu0818@163.com (M.Y.); liwuping1025@163.com (W.L.); zhihui@mail.hzau.edu.cn (Z.Z.); 2 Institute of Agricultural Applied Microbiology, Jiangxi Academy of Agricultural Sciences, Nanchang 330200, China; fljx99@163.com (L.F.); zhangfan9609@163.com (F.Z.); wucaiyunwy@163.com (C.W.); xuxueliang@126.com (X.X.); yaoyingjuan2008@163.com (Y.Y.)

**Keywords:** *M. incognita*, *P. aeruginosa*, nematocidal activity, stability, growth-promotion

## Abstract

*Meloidogyne incognita* is one of the most detrimental root-knot nematodes (RKNs) globally. The restricted application of chemical nematicides has resulted in an increasing inclination towards environment-friendly alternatives. In this study, a strain of *Pseudomonas aeruginosa* XR2-39, which was isolated from compost fermentation of edible fungus residue, exhibited effective biocontrol activity against *M. incognita*. In vitro experiments employing the fermentation filtrate of strain XR2-39 achieved high nematicidal efficacy of second-stage juveniles (J2s), resulting in corrected mortality rates of 97.12% and 100% after 24 h and 48 h, respectively. The fermentation filtrate also demonstrated a high relative hatching inhibition for egg masses (97.87%) and free eggs (100%). In addition, strain XR2-39 exhibited strong capabilities in indole-3-acetic acid (IAA) production (with a yield of 33.01 mg/L), siderophore production (with a yield of 71.45% unit), and phosphate solubilization (with a dissolved amount of 831.15 mg/L). Pot experiments indicated that the incubation of tomato roots with 20% fermentation broth led to an increase in fresh shoot weight, root weight, shoot length, root length, and stem diameter by 448.57%, 136.36%, 179.29%, 49.39%, and 57.14%, respectively, when compared to the water control. Moreover, the inoculation with 20% fermentation broth significantly decreased tomato root galls, resulting in a gall index of 37.00, which was significantly lower than that of the water-control treatment (77.50). Furthermore, the active compound in the fermentation filtrate remained stable within the pH range of 7.0 to 11.0, maintaining a corrected mortality rate of over 89.0%. It also demonstrated thermostability, as the boiled fermentation filtrate (treated at 120 °C for 2 h) showed a high corrected mortality rate against J2s. Additionally, the active substance displayed strong UV tolerance and storage stability. These characteristics of active compounds make strain XR2-39 a promising biocontrol agent for *M.* *incognita*.

## 1. Introduction

Plant-parasitic nematodes (PPNs) are destructive agricultural pathogens distributed worldwide, among which RKN is one of the most damaging pathogens, causing estimated annual economic losses exceeding 100 billion USD [1,2,3]. RKNs are predominantly found in tropical and subtropical regions and are capable of infecting over 3000 plant species globally, including vegetables, grains, weeds, and fruit plants [4,5]. Their transmission occurs through various pathways, such as contaminated soil, infected seeding, human activities, application of uncomposted manure, crop residue incorporation, and irrigation water [6,7,8]. Currently, chemical nematicides, such as ethoprop, fosthiazate, tioxazafen, and oxamyl, are widely used due to their efficiency [6]. However, their application is increasingly restricted in many countries owing to adverse environmental impacts, including non-target toxicity, disruption of the agricultural ecosystem, and risks to public health [9,10,11]. Consequently, there is a pressing need to develop environmentally sustainable alternatives for RKN management. Biological control agents derived from microorganisms or their active metabolites have attracted growing attention due to their eco-friendly properties and are regarded as viable components of sustainable nematode management strategies [12,13,14]. A growing number of bacterial and fungal strains have been identified as potential nematicidal agents, and their biocontrol efficacy has been extensively studied [14,15,16,17].

Plant growth-promoting rhizobacteria (PGPR), a ubiquitous group of microorganisms inhabiting the rhizosphere, are important biological resources for the control of *M. incognita* [18,19,20]. These microbes provide numerous beneficial functions to host plants, including the production of IAA and ethylene, secretion of chitinases, phosphate solubilization, siderophore production, nitrogen fixation, and protection against various pathogenic organisms [21]. PGPR has been shown to exert biocontrol effects against RKN through multiple mechanisms, such as parasitism, production of nematicidal compounds, intoxication of nematodes, induction of systemic resistance in plants, and enhancement of water and nutrient uptake [22,23]. Several PGPR strains have been demonstrated to have significant efficacy as biocontrol against RKNs [3,15,16,23,24,25,26,27,28,29,30,31]. For instance, *Burkholderia arboris* J211 exhibited strong nematicidal activity, causing 96.6% mortality of J2s after 24 h exposure, while also reducing root gall formation and promoting plant growth [28]. The cell-free filtrates of *Burkholderia* sp. JB-2 effectively reduced *M. incognita* population density in soil and suppressed egg mass development on tomato roots, concomitantly enhancing host plant growth [29]. Gao et al. [24] reported fewer and smaller root galls in tomato plants treated with *Bacillus cereus* S2. Burkett et al. [32] observed a reduction in *M. incognita* population in soil following application of *B. amyloliquefaciens* and *B. subtilis*. *Pseudomons fragi* Sneb1990 caused 76.8% J2 mortality after 48 h and inhibited egg hatching by 71.9% after 7 days, in addition to promoting plant growth [3]. Abd et al. [31] showed that *Serratia* spp. and *Pseudomonas* spp. significantly suppressed gall formation and enhanced the growth of *Luffa aegyptiaca* L. compared to untreated controls. *B. megaterium* was found to suppress soil RKN populations by modulating phosphate solubilization and mineralization capacity [33]. Despite the potential of many PGPR strains for RKN management, their practical application remains limited due to variability in activity and stability under different environmental conditions, such as soil texture, moisture, pH, and temperature [28]. Therefore, the discovery of novel antagonistic PGPR strains is essential to support large-scale production and integration into sustainable RKN control strategies. In the present study, we isolated a strain of *P. aeruginosa* XR2-39 exhibiting remarkable nematocidal activity and evaluated its effects on *M. incognita* in vitro. Furthermore, we also investigated the impact of the fermentation broth of XR2-39 on potted tomato plants grown in *M. incognita*-infested and non-infested soils under greenhouse conditions to assess its potential for both nematode control and plant growth promotion.

## 2. Materials and Methods

### 2.1. Plant Material and M. incognita Inoculum

Seeds of *Solanum lycopersicum* “Zhongza No.9”, a cultivar susceptible to *M. incognita*, were purchased from Zhongshu Seed Industry Technology Co., Ltd. (Beijingu, China). After surface sterilization with 10% sodium hypochlorite and rinsing thoroughly with sterile water, the seeds were germinated in a Petri dish lined with sterile filter paper and maintained at 60% relative humidity. Uniformly vigorous seeds were selected and sown into sterilized soil within a seedling tray, then cultivated in a greenhouse at 26 °C under a 16 h light/8 h dark photoperiod until they reached the four-true-leaf stage. Subsequently, the seedlings were divided into two groups, one for pot experiments and the other for *M. incognita* culture.

*M. incognita* was maintained on tomato roots and incubated in a growth chamber at a temperature of 26 °C under a 16 h light/8 h dark cycle for 45 days. Egg masses were harvested from infected roots and washed three times with sterile water. J2s were collected by hatching the egg masses through incubation at 26 °C for 3 days. Freshly hatched J2s were retrieved, disinfected with 1% sodium hypochlorite solution, and adjusted to a final concentration of 1000 individuals per milliliter for subsequent use.

### 2.2. Isolation of Biocontrol Bacterial Strains Against M. incognita

Bacterial strains were isolated from compost fermentation of edible fungus residue via the gradient dilution method using Luria–Bertani (LB) agar medium. Following incubation at 37 °C for 48 h, the strains were purified and stored at 4 °C for subsequent use.

### 2.3. Nematocidal Activity of Bacterial Fermentation Filtrates Against J2s of M. incognita In Vitro

All isolated bacterial strains were cultured separately in 20 mL of LB liquid medium at 37 °C for 20 h. The fermentation broth from each strain was diluted with sterilized water to a final density of 1.0 × 10^8^ cfu/mL [11]. The supernatant was collected by centrifuging at 12,000 rpm for 10 min. To evaluate the effect of bacterial strains on J2s mortality, the supernatant of each isolate was diluted twofold with sterile water and mixed with approximately 100 J2s in a sterile 24-well cell culture plate. Sterile water served as the control, and four replicates were established for each strain supernatant and the water control. After incubating in darkness at 25 °C for 24 h and 48 h, 3~4 drops of 15% NaCl were added to the well, and the samples were recovered for 30 min. Nematodes exhibiting straight bodies or no movement were considered dead [34]. The number of dead J2s was observed and recorded under a microscope, and the mortality of J2 was calculated. Both the mortality rate and corrected mortality rate of J2s were determined using the Equations (1) and (2), respectivly [35]:


(1) $$Mortality\;rate(\%)=\frac{Nd}{Nt}\times 100$$

(2) $$\mathrm{Corrected}\;\mathrm{mortality}\;\mathrm{rate}\;(\%)=\frac{Ms-Mc}{100-Mc}\times 100.$$

where *Nd* is the number of dead J2s; *Nt* is the total number of J2s; *Ms* is the mortality rate of J2s in the sample; and *Mc* is the mortality rate of J2s in the control.

### 2.4. Identification and Characterization of Plant Growth-Promoting (PGR) Traits of XR2-39

#### 2.4.1. Molecular Identification of Strain XR2-39 Against *M. incognita*

For molecular identification, genomic DNA of strain *P. aeruginosa* XR2-39, extracted using a bacterial genomic DNA extraction kit (TIANGEN, Beijing, China), was used as a template for polymerase chain reaction (PCR). Primers of 27F (5′-AGAGTTTGATCCTGGCTCAG-3′) and 1492R (5′-GGTTACCTTGTTACGACTT-3′) were employed for 16S rDNA gene amplification. The PCR amplification mixture consists of 2× PrimerSTAR mix (15.0 μL), 27F/1492R primers (1.0 μL each), genomic DNA (1.0 μL), and ddH_2_O (13.0 μL). The following amplification procedure was applied: initial denaturation at 94 °C for 4 min; followed by 29 cycles of denaturation at 94 °C for 10 s, annealing at 55 °C for 15 s, and extension at 72 °C for 1 min; and final extension at 72 °C for 10 min. The PCR product was sequenced by Sangon Biotech (Shanghai) Co., Ltd. (Shanghai, China), and the sequence analysis was conducted using the NCBI BLAST tool (http://www.ncbi.nlm.nih.gov/) (accessed on 9 September 2025) [11].

#### 2.4.2. Biochemical Characterization of Strain XR2-39

Bacterial strain XR2-39 was further characterized according to Bergey’s Manual of Determinative Bacteriology [36]. Subsequently, the strain cells were subjected to Gram staining for morphological identification [37]. Routine biochemical tests, including catalase activity, glycolysis, nitrate reduction, starch and gelatin hydrolysis, H_2_S production, citrate utilization, indole production, and Methyl Red (MR) and Voges-Proskauer (VP) tests, were performed on strain XR2-39 [38].

#### 2.4.3. IAA Production

IAA production by strain XR2-39 was determined using a colorimetric method as described by Tang and Bonner [39] with minor modifications. The strain was cultured in Nutrient Broth (NB) including L-tryptophan (100 mg/L) and incubated at 30 °C (180 rpm) for 7 days. The supernatant was collected by centrifugation at 10,000 rpm for 10 min. A volume of 2 mL of Salkowaski reagent (50 mL of 35% perchloric acid mixed with 1 mL of 0.5 M FeCl_3_) was added to 2 mL of supernatant, and the mixture was incubated in the dark at room temperature for 30 min. Absorbances were measured at 530 nm. The IAA concentration was determined based on a standard curve of IAA [40].

#### 2.4.4. Phosphate Solubilization

The strain XR2-39 was cultured in 100 mL of phosphorus-solubilizing culture medium (containing 1.0 g glucose, 0.5 g Ca_3_(PO4)_2_, 0.5 g MgCl_2_, 0.025 g MgSO_4_·7H_2_O, 0.02 g KCl, and 0.001 g (NH_4_)_2_SO_4_) at 30 °C with shaking at 180 rpm for 7 days. The supernatant was collected by centrifugation at 4 °C (10,000 rpm, 10 min). The concentration of soluble phosphorus was determined using the antimony molybdenum anti-colorimetric method [41].

#### 2.4.5. Siderophore Production

The strain XR2-39 was cultured in 100 mL of siderophore-inducing medium (containing 10.0 g glucose, 2.0 g peptone, 0.05 g MgSO_4_·7H_2_O, and 0.05g CaCl_2_, 20 mM Pipes buffer) at 30 °C with shaking at 180 rpm for 7 days. The supernatant was collected by centrifugation at 4 °C (10,000 rpm, 10 min). Subsequently, 200 μL of supernatant was mixed with 200 μL of CAS reagent (containing 0.2 M 5-sulfosalicyclic acid). Absorbance was measured at 630 nm after incubation at 26 °C for 20 min [28]. The percent of siderophore was intended in terms of % of siderophore units by means as follows [28]:

(3) $$\mathrm{Siderophore}\;\mathrm{Unit}\;(\%)\;=\;\frac{A_{c}-A_{s}}{Ac}\times 100,$$

where *Ac* is the absorbance of the control at 630 nm, and *As* is the absorbance of the sample at 630 nm.

### 2.5. Effect of Strain XR2-39 Fermentation Filtrate on J2s, Egg Mass, and Free Egg Hatching of M. incognita

#### 2.5.1. Effects of Strain XR2-39 Fermentation Filtrates on J2s of *M. incognita* In Vitro

A total of 100 J2s were added to each well of a sterile 24-well cell culture plate containing 500 μL of fermentation filtrate at concentrations of 100%, 20%, 10%, and 5%, respectively. Distilled water served as the control treatment, and four replicates were established for each concentration. After incubating in darkness at 25 °C for 24 h and 48 h, the J2s mortality rates were determined in Section 2.3.

#### 2.5.2. Effects of Strain XR2-39 Fermentation Filtrates on Egg Masses In Vitro

Mature egg masses of uniform size and similar color were selected and disinfected with 0.5% sodium hypochlorite for 3 min, followed by rinsing with sterile water three times to ensure complete removal of residual sodium hypochlorite from the surface. To evaluate the in vitro biocontrol efficiency of strain XR2-39 fermentation filtrate on egg masses, the strain was cultured in LB liquid medium, and the resulting filtrate was diluted to a density of 1.0 × 10^8^ cfu/mL with sterilized water. Three sterilized egg masses were placed in a 50 mL sterile centrifuge tube and exposed to fermentation filtrate at concentrations of 100%, 20%, 10%, and 5%, respectively. Sterile water treatment was set as the control. All treatments were performed in quadruplicate and incubated at 25 °C. After 1, 2, 3, 4, and 5 days, the number of hatched J2s was recorded under a microscope. The relative hatching inhibition was subsequently calculated as follows:

(4) $$\mathrm{Hatching}\;\mathrm{rate}\;(\%)\;=\frac{NJ2s}{NJ2s+Negg}\times 100,$$

(5) $$\mathrm{Relative}\;\mathrm{hatching}\;\mathrm{inhibition}\;(\%)=\frac{Hc-Hs}{Hc}\times 100.$$

where *NJ2s* is the number of J2s; *Negg* is the number of eggs; *Hc* is the hatching rate of the control; and *Hs* is the hatching rate of the sample.

#### 2.5.3. Effects of Strain XR2-39 Fermentation Filtrates on Free Eggs In Vitro

*M. incognita* free eggs were collected from infected tomato roots following the method described by Hussey and Barker [42]. The free eggs were suspended in distilled water and adjusted to a concentration of approximately 1000 eggs/mL. Approximately 100 eggs were exposed to each of the 100%, 20%, 10%, or 5% fermentation filtrate, respectively, with eggs treated with distilled water serving as the control. All treatments were conducted in quadruplicate and incubated at 25 °C. The number of hatched J2s was recorded under a microscope at 1, 2, 3, 4, and 5 day intervals. The relative hatching inhibition was calculated according to Equation (5).

### 2.6. Effect of Strain XR2-39 Fermentation Broth on Growth of Tomato Plants in Pot Experiment

To evaluate the effect of strain XR2-39 on tomato plant growth, a pot experiment was conducted using tomato seedlings with four true leaves. Each seedling was transplanted into a pot containing 450 g of sterile matrix soil. Three days after transplantation, the seedlings were divided into five groups and subjected to root irrigation with 50 mL of 100%, 20%, 10%, or 5% fermentation filtrate, respectively, while the control group received 50 mL of sterile water. Root irrigation was performed every five days for a total of three applications. Each treatment consisted of three replicates, with 10 plants per replicate. Plant height, fresh shoot weight, root weight, stem diameter, and root length were measured at 15 days post-inoculation (dpi), following the final root irrigation.

### 2.7. Biocontrol of Strain XR2-39 Fermentation Broth Against M. incognita on Tomato Plants in a Pot

To evaluate the biocontrol efficacy of strain XR2-39 against nematode infection, a pot experiment was conducted using tomato seedlings with four true leaves. Each seedling was transplanted into a pot containing 450 g of sterile matrix soil. Three days after transplantation, seedlings were subjected to root irrigation with 50 mL of 20% XR2-39 fermentation filtrate. Treatments receiving 50 mL of sterile water or 50 mL of abamectin (1.0 mg/L) served as negative and positive controls, respectively. Each treatment consisted of three replicates, with 10 plants per replicate. Three days later, a small hole was made in the soil at a depth of 1 cm and located 2~3 cm from the root, into which 1 mL of a suspension containing 400 J2s was injected. At 40 dpi, plants were harvested, and roots were gently rinsed under running water. Plant height, fresh shoot weight, root weight, stem diameter, and root length were recorded. Concurrently, the root-knot nematode infestation level, root-knot index, and biocontrol efficacy were determined according to the method described by Bridge and Page [43]. Briefly, root-knot severity was classified into the following five grades: (0) no galls; (1) 1~15% of the root system affected; (2) 16~25% affected; (3) 26~50% affected; (4) 51~75% affected; and (5) more than 76% affected. The root-knot index and biocontrol efficacy were calculated as follows:

(6) $$\mathrm{Root}\text{-}\mathrm{knot}\;\mathrm{index}\;(\%)\;=\;\frac{\sum \left(Ndis\times Vcd\right)}{Nt\times Vhd}\times 100,$$

(7) $$\mathrm{Biocontrol}\;\mathrm{effect}\;(\%)=\left(1-\frac{RIs}{RIc}\right)\times 100.$$

where *Ndis* is the number of diseased plants; *Nt* is the number of total investigated plants; *Vcd* is the value of the corresponding disease level; *Vhd* is the value of the highest investigated disease level; *RIs* is the root-knot of the sample; and *RIc* is the root-knot of the control.

### 2.8. Effect of pH, Temperature, and UV Irradiation and Storage on Stability of Strain XR2-39 Fermentation Filtrate

The pH stability was determined by incubating XR2-39 fermentation filtrate at pH values of 3.0, 4.0, 5.0, 6.0, 7.0, 8.0, 9.0, 10.0, and 11.0 for 12 h at 25 °C. After incubation, the pH was adjusted to 7.0 using 1.0 mol/L HCl or 1.0 mol/L NaOH prior to activity assessment. Thermostability was assessed by incubating fermentation filtrate at −20, 0, 20, 40, 60, 80, 100, and 121 °C for 2 h, respectively. The tolerance of UV irradiation was determined by exposing the filtrate to UV irradiation (15 W) at a distance of 15 cm from the source for 1, 2, 3, 4, 5, and 6 h. To evaluate storage stability, the fermentation filtrate was stored at 4 °C and 25 °C for 3, 7, 14, 28, and 31 days, respectively. Following centrifugation at 10,000 rpm for 5 min, the supernatant was filtered with a 0.22 μm membrane filter. The corrected mortality rate was measured in Section 2.3.

## 3. Results

### 3.1. Isolation of Biocontrol Bacterial Strain XR2-39 Against M. incognita

**Figure 1:**
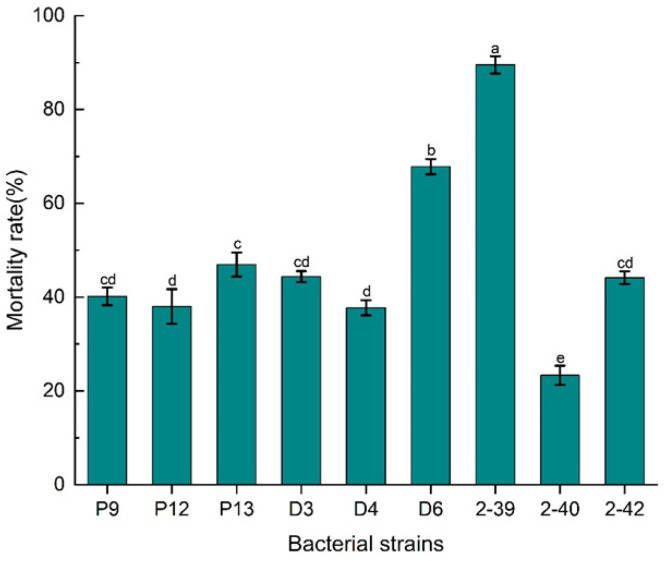
The mortality rate of the bacterial strains on J2s of *M. incognita*. Different lowercase letters indicate significant differences among strains (*p* < 0.05).

A total of nine bacterial strains exhibiting nematocidal activities against *M. incognita* were isolated from the compost fermentation of edible fungus residue. Preliminary characterization revealed that strain XR2-39 displayed the highest nematocidal activity, reaching 89.53% (Figure 1), and was therefore selected for identification and further biocontrol evaluation. Microscope observation showed that XR2-39 cells were rod-shaped, and Gram staining indicated that XR2-39 is a Gram-negative bacterium. On LB agar plates, XR2-39 cells formed rufous, moist colonies with flat and irregular margins.

### 3.2. Identification and Characterization of Plant Growth-Promoting (PGR) Traits of XR2-39

**Figure 2:**
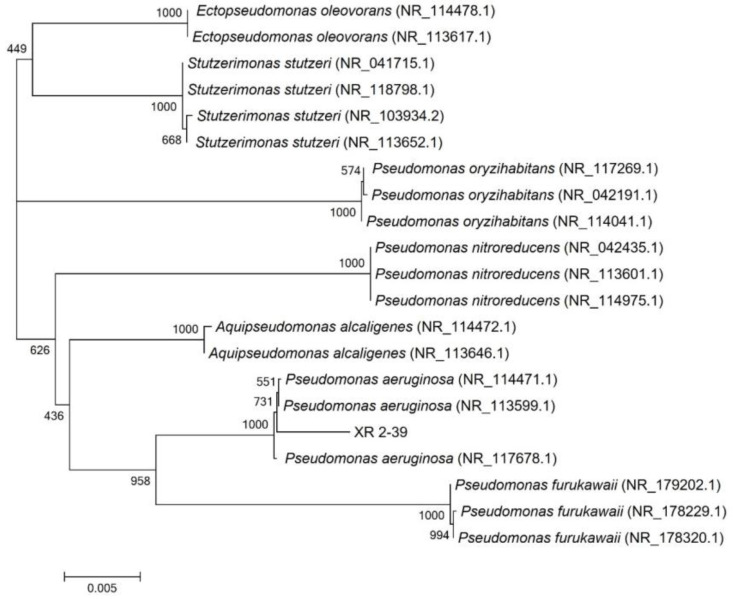
Phylogenetic tree constructed with the neighbor-joining method based on 16S rDNA genes.

The biochemical characterization of XR2-39 showed positive reactions for catalase activity, nitrate reduction, starch hydrolysis, gelatin hydrolysis, and citrate utilization, while exhibiting negative results for Gram staining, glycolysis, indole production, MR test, VP test, and H_2_S production. Furthermore, BLAST (2.17.0) analysis of the 16S rDNA gene sequence of XR2-39 against the NCBI database revealed that the strain shares 100% sequence similarity with the 16S rDNA gene of *Pseudomonas aeruginosa*. The maximum likelihood phylogenetic tree based on 16S rDNA gene sequences further confirmed the close phylogenetic relationship between strain XR2-39 and members of the species of *P. aeruginosa* (Figure 2). Based on the biochemical and molecular analysis, strain XR2-39 was identified as *P. aeruginosa* XR2-39 and was deposited in the Guangdong Microbial Culture Collection Center (GDMCC No: 64092).

Furthermore, strain XR2-39 exhibited a strong capacity to produce IAA, with a yield of 33.01 mg/L. Phosphate solubilization analysis revealed that XR2-39 could dissolve inorganic phosphorus, achieving a solubilized concentration of 831.15 mg/L. In addition, XR2-39 demonstrated high siderophore production, reaching 71.45% (Table 1).

### 3.3. Nematocidal Activity of Strain XR2-39 Fermentation Filtrate Against M. incognita J2s In Vitro

**Figure 3:**
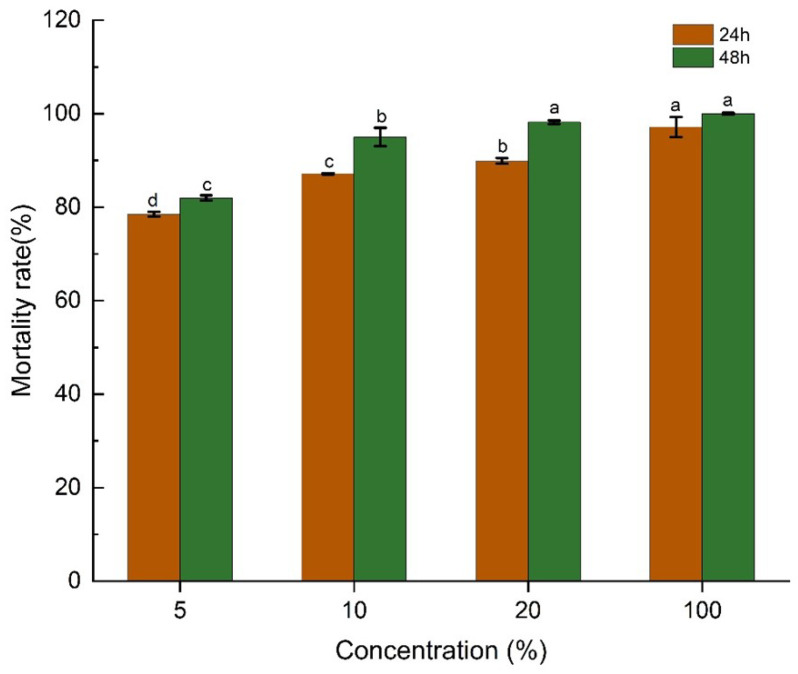
The mortality rates of different concentrations of XR2-39 fermentation filtrate at 24 h and 48 h. Different letters in the bar with the same color indicate a significant difference (*p* < 0.05).

To evaluate the nematocidal potential of the fermentation filtrate of strain XR2-39, 100%, 20%, 10%, and 5% dilutions were tested for their effects on J2 mortality. Results showed that all concentrations exhibited nematocidal activity against J2s, with the corrected mortality rate increasing over time and with high concentration. After 24 h and 48 h of exposure, fermentation filtrate demonstrated strong nematocidal activity, resulting in corrected mortality rates of 97.12% and 100%, respectively. Lower concentrations induced relatively lower mortality. For instance, 20% filtrate caused corrected mortality rates of 89.87% and 98.18% at 24 h and 48 h, respectively. The 10% filtrate resulted in 87.14% and 95.04%, while the 5% filtrate yielded 78.52% and 81.97% of corrected mortality at 24 h and 48 h, respectively (Figure 3).

In addition, microscopic observation revealed that the fermentation filtrate damaged the internal organs and body wall of *M. incognita* J2s, resulting in the production of vacuoles and cuticle degradation. However, the sterile water did not induce these symptoms in *M. incognita* J2s (Supplementary Figure S1).

### 3.4. Effect of Strain XR2-39 Fermentation Filtrate on Egg Mass and Free Egg Hatching of M. incognita

Regarding the effect of XR2-39 on egg mass and free egg hatching, a decrease in the relative hatching inhibition was observed with decreasing fermentation filtrate concentration, whereas an increase in relative hatching inhibition was observed with prolonged incubation time. The highest hatching inhibition of 97.87% for egg masses and 100% for free eggs was recorded after 1 day of incubation with 100% fermentation filtrate. No J2s of *M. incognita* were detected in the 100% fermentation filtrate after 5 days of incubation, likely due to the degradation of *M. incognita* J2s. The 20% fermentation filtrate also exhibited high relative hatching inhibition, reaching 96.52% for egg masses and 75.93% for free eggs, followed by the 10% filtrate, which resulted in relative hatching inhibition of 87.15% and 70.58% for egg masses and free eggs, respectively (Table 2).

### 3.5. Effect of Strain XR2-39 Fermentation Broth on Growth of Tomato Plants in Pot Experiment

Inoculation with 20% fermentation broth on tomato roots increased fresh shoot weight, root weight, shoot length, root length, and stem diameter by 448.57%, 136.36%, 179.29%, 49.39%, and 57.14%, respectively, compared to the water control. Treatment with 100% fermentation broth also promoted longer shoot and root development by 71.00% and 96.97%, increasing fresh shoot weight and stem diameter by 277.14% and 57.14%, respectively. The 10% fermentation broth increased shoot length and root length by 56.14% and 74.85%, respectively. Among all tested parameters, only stem diameter showed a significant difference from the water control in plants treated with 5% fermentation broth (*p* < 0.05) (Table 3).

### 3.6. Effect of Strain XR2-39 Fermentation Broth Against M. incognita in Pot Plants

Results of the plant growth-promoting experiment indicated that the 20% fermentation broth exhibited the most effective growth-promoting activity. Therefore, this concentration was selected for further evaluation of the suppressive effect on *M. incognita* in pot experiments. Treatment with 20% XR2-39 fermentation significantly reduced *M. incognita* infection in tomato plants, as evidenced by fewer and smaller root galls and enhanced root system development compared to the water control. Both abamectin and XR2-39 fermentation broth treatments resulted in improved root health compared to the control. The gall index of the XR2-39 fermentation broth treatment was 37.00, which was significantly lower than that of the water control treatment (77.50), though higher than that of the abamectin treatment (29.00). The nematicidal control efficiency of the XR2-39 fermentation broth treatment was 52.44%, which was lower than that of the abamectin (62.36%) (Table 4).

Meanwhile, compared with the water control treatment, the XR2-39 fermentation broth treatment increased fresh shoot weight, root length, and stem diameter by 32.42%, 23.81%, and 13.51%, respectively. However, there were no significant differences in measured plant parameters between XR2-39 fermentation broth and abamectin treatments, except for fresh shoot weight (Table 4).

### 3.7. Effect of pH, Temperature, and Ultraviolet Irradiation and Storage on Stability of Strain XR2-39 Fermentation Filtrate

**Figure 4:**
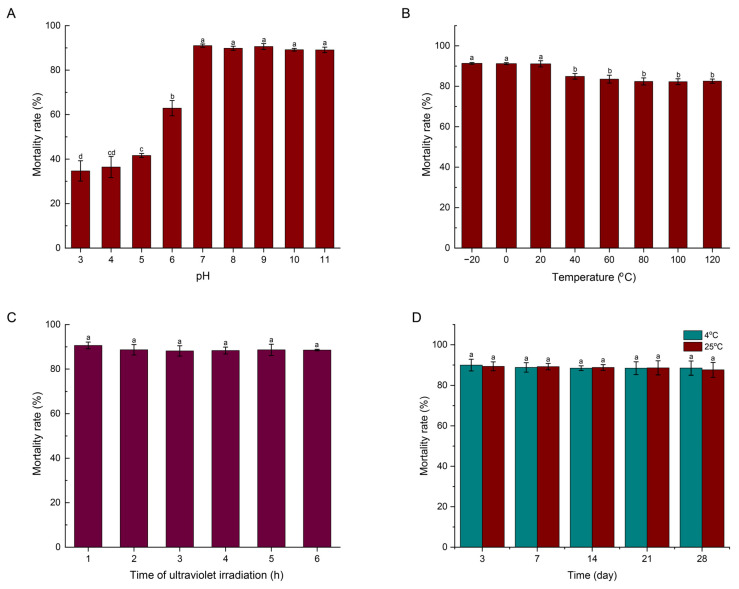
The pH (**A**), temperature (**B**), UV irradiation (**C**), and storage (**D**) effects on the stability of XR2-39 fermentation filtrate. Different letters in the bar with the same color indicate a significant difference (*p* < 0.05).

The analysis of the pH effect on corrected mortality revealed that the XR2-39 fermentation filtrate was stable within the pH range of 7.0 to 11.0, with the corrected mortality remaining above 89.0%. However, the nematocidal activity decreased as the pH declined, resulting in corrected mortalities of only 34.68%, 36.43%, and 41.64% mortality at pH levels of 3.0, 4.0, and 5.0, respectively. Temperature stability analysis demonstrated that storage at −20 °C, 0 °C, and 20 °C had no significant effect on corrected mortality induced by XR2-39 fermentation filtrate. Moreover, high levels of nematocidal activity were maintained even at elevated temperatures; for instance, a mortality rate of 82.27% was retained after exposure to 100 °C for 2 h. In addition, ultraviolet irradiation duration showed no significant effect on nematocidal activity, with corrected mortality rates of 90.62%, 88.65%, 88.15%, 88.32%, 88.63%, and 88.53% observed after 1, 2, 3, 4, 5, and 6 h of exposure, respectively. Furthermore, after storage at 4 °C and 25 °C for 28 d, the filtrate still exhibited high mortality rates of 88.49% and 87.63%, respectively. Collectively, these results indicate that the XR2-39 fermentation filtrate possesses favorable properties, including alkali tolerance, thermal stability, UV irradiation resistance, and long-term storage stability (Figure 4).

## 4. Discussion

Plant-parasitic nematodes cause severe damage to agriculture worldwide [2]. Although chemical nematocidal agents provide effective solutions for mitigating substantial crop losses, their adverse effects on the environment and human health restrict widespread application [44]. Therefore, researchers have sought eco-friendly biological control agents to manage nematode infestations over recent decades, with particular focus on antagonistic microorganisms. A number of fungi and bacterial strains have been identified as effective nematicides [16,26,45]. Specifically, PGPR are beneficial microbes that colonize the rhizosphere and plant roots, promoting plant growth and enhancing resistance to pathogens [46]. Due to their potential for controlling PPNs and promoting plant growth, PGPR have gained increasing attention as promising alternatives to chemical controls for managing PPNs [23,28]. Among PGPR, *Bacillus* sp. and *Pseudomonas* sp. have been reported to possess the ability to control *M. incognita*. In this study, strain *P. aeruginosa* XR2-39, isolated from compost fermentation of edible fungus residue, exhibited high nematicidal activity against *M. incognita*. The fermentation filtrate of strain XR2-39 caused significant mortality of J2s and reduced egg hatching of *M. incognita*. However, both mortality and hatching inhibition were directly influenced by the fermentation concentration and exposure time. Our results revealed that all tested concentrations of the fermentation filtrate exhibited high nematocidal activity against *M. incognita*. The highest efficacy was observed with 100% XR2-39 fermentation filtrate, which achieved 100% corrected J2 mortality at 48 h. This result was consistent with the findings of Hu et al. [23], who reported that the J2s were killed in the culture filtrate of Bv-DS1 after 48 h of treatment. Hussain et al. [47] also observed a corresponding increase in mortality with rising concentration, with J2 mortality reaching 52.47% at 50% concentration and increasing to 72.10% at 100% concentration after 48 h treatment; mortality further increased to 95.90% after 72 h treatment at 100% concentration. In vitro experiments showed *B. licheniformis* JF-22, isolated from the tomato rhizosphere soil, achieved 77% inactivation of *M. incognita* J2s after 24 h and 89% after 48 h of incubation [25]. Gao et al. [24] demonstrated that the fermentation filtrate of *B. cereus* strain S2 caused 90.96% mortality in *M. incognita* J2s. Similar antagonistic effects have been observed in certain *Pseudomonas* strains, which displayed biocontrol activities against *M. incognita*. Zhai et al. [48] reported that most *M. incognita* J2s became immobile when incubated with *P. putida* 1A00316 for 72 h. Zhao et al. [37] found that the mortality rate of *P. fluorescens* (Sneb 825) increased with prolonged exposure time, reaching a maximum of 80.00% at 96 h.

Our results also showed that all concentrations of XR2-39 fermentation filtrate significantly inhibited hatching of both free eggs and egg masses, with the highest hatching inhibition observed at 100% concentration after 2 days. This was consistent with the findings of Rao et al. [49], who reported that *B. subtilis* significantly reduced the egg hatching across various concentrations and exposure durations, achieving a maximum inhibition of 94.65% at 100% concentration after 5 days. Hussain et al. [47] demonstrated that egg hatching was progressively inhibited, and no juveniles emerged as incubation time increased. *B. cereus* strain Bc-cm103 has been reported as a biocontrol agent against *M. incognita* hatching eggs, with the lowest egg hatching rate recorded in treatment with supernatant (9.99%), followed by 10% supernatant (13.43%) [50]. Hatching inhibition was also found to be directly related to the exposure duration in *Pseudomonas* spp. [31]. Notably, no egg hatching was observed after 48 h of treatment with 100% XR2-39 filtrate. These results suggested that strain XR2-39 fermentation filtrate may contain nematocidal metabolites that are toxic to *M. incognita*. Further studies are needed to identify the active substance responsible for its antagonistic effects against *M. incognita*.

The pot experiment demonstrated a significant reduction in *M. incognita* infection in tomato plants, which may contribute to the suppression of *M. incognita* infection at early stages, resulting in a subsequent reduction in root gall formation and egg mass accumulation in the tomato root system following treatment with XR2-39. In addition to its effect on reducing tomato susceptibility to *M. incognita*, the pot experiment also revealed that strain XR2-39 exhibited significant plant growth-promoting activities. Although the mechanism of action of strain XR2-39 was not elucidated in these experiments, it may be hypothesized that strain XR2-39 metabolites inhibited egg hatch and caused mortality of *M. incognita* J2s. Concurrently, systemic resistance in plants was induced by strain XR2-39 colonizing the rhizosphere, thereby enhancing the host’s ability to resist nematode invasion and subsequent infection. Abd El-Aa et al. [31] reported that application of *Serratia* sp. and *Pseudomonas* spp. resulted in a high level of J2s mortality and a beneficial effect on plant growth. Enhanced growth associated with PGPR application has also been observed in tomato [44], cucumber [51], rice [52], barley [53], pepper [54], and melon [55]. It is well established that PGPR can produce the phytohormone auxin to promote plant growth [56,57], and their growth-promoting effects are mediated through multiple mechanisms, such as acquisition of limiting nutrients, phosphate solubilization [58], siderophore production [59], and nitrogen fixation [60]. The PGP traits of strain XR2-39 were evaluated, revealing outstanding abilities in phosphate solubilization, IAA production, and siderophore synthesis, with yields reaching 831.15 mg/L, 33.01 mg/L, and 71.45%, respectively. Islam et al. [51] demonstrated that *P. stutzeri*, *B.subtilis*, *Stenotrophomonas maltophilia*, and *B. amyloliquefaciens* isolated from cucumber rhizosphere produced IAA levels at concentrations ranging from 26.78 to 51.28 μg/mL, and treatment with these strains significantly promoted the cucumber growth. Furthermore, Singh et al. [61] showed that *B. arboris* CSRS12 enhanced lateral root development in mung bean through phosphate solubilization and siderophore production. Therefore, in this study, the plant growth-promoting effect may be attributed to two factors: first, the reduction in root damage caused by *M. incognita* due to XR2-39 activity and second, the strain’s capacity to produce IAA and siderophores, as well as to solubilize inorganic phosphate. These results indicate that XR2-39 plays an important role in promoting plant growth and biocontrol activity against *M. incognita*. Further studies are needed to elucidate the underlying mechanisms of plant growth promotion and biocontrol activity by strain XR2-39.

Moreover, the stability of the active abundances produced by biocontrol strains plays a crucial role in disease prevention and significantly influences their practical application [62]. External environmental conditions have a substantial impact on the activity and stability of biocontrol strains. Substances produced by *B. altitudinis* AMCC 1040 exhibited no difference in nematicidal activity when exposed to pH 2.0~6.0, resulting in J2 mortality exceeding 90%; however, the activity declined sharply and nearly disappeared under alkaline conditions [63]. In contrast, the fermentation filtrate of XR2-39 was stable within the pH range of 7.0 to 11.0, maintaining nematocidal activity with mortality rates above 89.0%, whereas its efficacy decreased at pH 3.0~5.0, with mortality reduced to 34.68%~41.64%. In addition, the fermentation filtrate of XR2-39 demonstrated excellent thermostability, as boiled filtrate still induced high J2 mortality even after treatment at 120 °C, which is consistent with the findings reported by Ye et al. [63]. Furthermore, the fermentation filtrate of XR2-39 exhibited strong UV tolerance and long-term storage stability. These favorable characteristics of the active compounds suggest that strain XR2-39 has strong potential as a biocontrol agent against *M. incognita*.

## 5. Conclusions

Strain XR2-39 was isolated from a compost fermentation of edible fungus residue and exhibited pronounced nematocidal activity, egg hatching inhibition, and plant growth-promoting effects. The strain demonstrated outstanding capabilities in phosphate solubilization, siderophore, and IAA production. Furthermore, the activity substances produced by XR2-39 showed alkali stability, high thermostability, strong UV tolerance, and long-term storage ability. However, further research is needed to identify the biological molecules responsible for its antagonistic effects, as well as to elucidate the mechanisms underlying its biocontrol activity and plant growth promotion.

## Figures and Tables

**Table 1:** Physiochemical characteristics and plant growth-promoting (PGP) properties of strain XR2-39.

Biochemical Test	Test Result
Gram staining	−
Catalase activity	+
Glycolysis	−
Nitrate reduction	+
Starch hydrolysis	+
Gelatin hydrolysis	+
Indole test	−
Citrate utilization	+
MR test	−
VP test	−
H_2_S production	−
IAA production (mg/L)	33.01 ± 1.36
Phosphate Solubilization (mg/L)	831.15 ± 21.40
Siderophore production (%)	71.45 ± 2.51

**Table 2:** The egg hatching inhibition of different concentrations of XR2-39 fermentation filtrate.

Treatment	Egg Mass (%)				
	1 d	2 d	3 d	4 d	5 d
100% Fermentation filtrate	97.87 ± 2.71 a	100 ± 0.001 a	100 ± 0.00 a	100 ± 0.00 a	100 ± 0.00 a
20% Fermentation filtrate	73.91 ± 6.07 b	91.26 ± 2.60 b	90.30 ± 4.19 b	95.10 ± 2.45 a	96.52 ± 3.25 ab
10% Fermentation filtrate	59.82 ± 4.52 c	77.11 ± 4.21 c	83.64 ± 8.51 b	87.48 ± 8.47 b	87.15 ± 1.13 b
5% Fermentation filtrate	43.02 ± 1.40 d	64.28 ± 6.28 d	65.72 ± 6.47 c	63.8 ± 1.19 c	65.47 ± 11.00 c
	**Free egg (%)**				
100% Fermentation filtrate	100 ± 0.00 a	99.13 ± 1.73 a	100 ± 0.00 a	100 ± 0.00 a	100 ± 0.00 a
20% Fermentation filtrate	47.16 ± 5.80 b	56.60 ± 8.35 b	70.82 ± 5.50 b	75.11 ± 5.11 b	75.93 ± 8.44 b
10% Fermentation filtrate	36.29 ± 3.77 c	38.22 ± 7.02 c	57.16 ± 2.02 c	68.70 ± 6.29 b	70.58 ± 7.59 b
5% Fermentation filtrate	30.18 ± 6.49 c	36.20 ± 1.17 c	49.62 ± 6.99 c	51.73 ± 9.35 c	48.68 ± 5.99 c

Data were presented as mean ± SE of the mean; values followed by the same letter indicate a significant difference (*p* < 0.05; *n* = 4) between treatments according to the Tukey test.

**Table 3:** Effects of different concentrations of XR2-39 fermentation broth on growth parameters of tomato plants.

Treatment	Shoot Length (cm)	Fresh Shoot Weight (g)	Root Weight (g)	Stem Diameter (cm)	Root Length (cm)
100% Fermentation	11.97 ± 0.39 b	1.32 ± 0.37 b	0.15 ± 0.06 b	0.33 ± 0.03 a	6.50 ± 2.55 a
20% Fermentation	19.55 ± 1.39 a	1.92 ± 1.57 a	0.26 ± 0.03 a	0.33 ± 0.01 a	4.93 ± 1.01 abc
10% Fermentation	10.93 ± 1.04 b	0.55 ± 0.43 c	0.13 ± 0.01 b	0.26 ± 0.02 b	5.77 ± 1.45 ab
5% Fermentation	7.72 ± 0.58 c	0.37 ± 0.15 c	0.11 ± 0.04 b	0.24 ± 0.03 b	4.00 ± 0.70 bc
Water control	7.00 ± 1.39 c	0.35 ± 0.1 c	0.11 ± 0.05 b	0.21 ± 0.03 c	3.30 ± 1.19 c

Data were presented as mean ± SE of the mean; values followed by the same letter indicate a significant difference (*p* < 0.05; *n* = 10) between treatments according to the Tukey test.

**Table 4:** Effects of different treatments on parameters of tomato plants and *M. incognita*.

Treatment	Shoot Length (cm)	Fresh Shoot Weight (g)	Root Length (cm)	Root Weight (g)	Stem Diameter (cm)	Root-Knot Index	Biocontrol Effect (%)
Abamectin	30.09 ± 2.20 a	7.59 ± 0.56 a	7.18 ± 1.10 ab	0.50 ± 0.11 a	0.44 ± 0.04 a	29.00 ± 0.05 c	62.36
20% XR2-39 fermentation	30.48 ± 0.65 a	6.29 ± 0.18 b	8.06 ± 0.67 a	0.48 ± 0.07 a	0.42 ± 0.02 a	37.00 ± 0.08 b	52.44
Water control	29.17 ± 0.62 a	4.75 ± 0.16 c	6.51 ± 0.67 b	0.43 ± 0.08 a	0.37 ± 0.01 b	77.50 ± 0.05 a	-

Data were presented as mean ± SE of the mean; values followed by the same letter indicate a significant difference (*p* < 0.05; *n* = 30) between treatments according to the Tukey test.

## Data Availability

The original contributions presented in this study are included in the article/Supplementary Material. Further inquiries can be directed to the corresponding authors.

## Supplementary Materials

The following supporting information can be downloaded at: https://www.mdpi.com/article/10.3390/microorganisms14010005/s1, Figure S1: Morphological characteristics of *M. incognita* J2s observed under an optical microscope. (A) sterile water treatment, (B) fermentation filtrate of strain XR2-39 treatment.

## References

[1] Elling A.A. (2013). Major emerging problems with minor *Meloidogyne* species. Phytopathology.

[2] Jones J.T., Haegeman A., Danchin E.G.J., Gaur H.S., Helder J., Jones M.G.K., Kikuchi T., Manzanilla-López R., Palomares-Rius J.E., Wesemael W.M.L. (2013). Top 10 plant-parasitic nematodes in molecular plant pathology. Mol. Plant Pathol..

[3] Wang S., Fan H., Zhao D., Zhu X., Wang Y., Liu X., Liu D., Duan Y., Chen L. (2021). Multifunctional efficacy of the nodule endophyte *Pseudomonsa fragi* in stimulating tomato immune response against *Meloidogyne incognita*. Biol. Control.

[4] Abad P., Favery B., Rosso M.N., Castagnone-Sereno P. (2003). Root-knot nematode parasitism and host response: Molecular basis of a sophisticated interaction. Mol. Plant Pathol..

[5] Mitiku M. (2018). Plant-parasitic nematodes and their management: A review. Agric. Res. Technol..

[6] Zhao Z., Wang L., Khan R.A.A., Song X., Najeeb S., Zhao J., Yang Y., Ling J., Mao Z., Jiang X. (2023). *Methylorubrum rhodesianum* M520 as a biocontrol agent against *Meloidogyne incognita* (Tylenchida: Heteroderidae) J2s infecting cucumber roots. J. Appl. Microbiol..

[7] Kiewnick S., Sikora R.A. (2006). Evaluation of *Paecilomyces lilacinus* strain 251 for the biological control of the northern root-knot nematode *Meloidogyne hapla* Chitwood. Nematology.

[8] Moens M., Perry R.N., Starr J.L., Perry R.N., Moens M., Starr J.L. (2009). Meloidogyne Species—A Diverse Group of Novel and Important Plant Parasites.

[9] Schneider S.M., Rosskopf E.N., Leesch J.G., Chellemi D.O., Bull C.T., Mazzola M. (2003). United states department of agriculture-agricultural research service research on alternatives to methyl bromide: Pre-plant and post-harvest. Pest Manag. Sci..

[10] Forghani F., Hajihassani A. (2020). Recent advances in the development of environmentally benign treatments to control root-knot nematodes. Front. Plant Sci..

[11] Yin N., Liu R., Zhao J.L., Khan R.A.A., Mao Z. (2021). Volatile organic compounds of *Bacillus cereus* strain Bc-cm103 exhibit fumigation activity against *Meloidogyne incognita*. Plant Dis..

[12] Abd-Elgawad M.M., Askary T.H. (2018). Fungal and bacterial nematicides in integrated nematode management strategies. Egypt. J. Biol. Pest Control.

[13] Kanwar R.S., Patil J.A., Yadav S. (2021). Prospects of using predatory nematodes in biological control for plant parasitic nematodes—A review. Biol. Control.

[14] Khan R.A.A., Najeeb S., Mao Z., Ling J., Yang Y., Li Y., Xie B. (2020). Bioactive secondary metabolites from *Trichoderma* spp. against phytopathogenic bacteria and root-knot nematode. Microorganisms.

[15] Geng C., Nie X., Tang Z., Zhang Y., Lin J., Sun M., Peng D. (2016). A novel serine protease, Sep1, from *Bacillus firmus* DS-1 has nematicidal activity and degrades multiple intestinal-associated nematode proteins. Sci. Rep..

[16] Kamalanathan V., Sevugapperumal N., Nallusamy S. (2023). Antagonistic bacteria *Bacillus velezensis* VB7 possess nematicidal action and induce an immune response to suppress the infection of root-knot nematode (RKN) in tomato. Genes.

[17] Kim J.-H., Lee B.-M., Lee H.C., Choi I.-S., Koo K.-B., Son K.-H. (2024). Antagonistic efficacy of symbiotic bacterium *Xenorhabdus* sp. SCG against *Meloidogyne* spp.. J. Microbiol. Biotechnol..

[18] Paul D., Lade H. (2014). Plant-growth-promoting rhizobacteria to improve crop growth in saline soils: A review. Agron. Sustain. Dev..

[19] Bhattacharyya P.N., Jha D.K. (2012). Plant growth-promoting rhizobacteria (PGPR): Emergence in agriculture. World J. Microbiol. Biotechnol..

[20] Viljoen J.J.F., Labuschagne N., Fourie H., Sikora R.A. (2019). Biological control of the root-knot nematode *Meloidogyne incognita* on tomatoes and carrots by plant growth-promoting rhizobacteria. Trop. Plant Pathol..

[21] Borah B., Ahmed R., Hussain M., Phukon P., Wann S.B., Sarmah D.K., Bhau B.S. (2018). Suppression of root-knot disease in *Pogostemon cablin* caused by *Meloidogyne incognita* in a rhizobacteria mediated activation of phenylpropanoid pathway. Biol. Control.

[22] Cetintas R., Kusek M., Fateh S.A. (2018). Effect of some plant growth-promoting rhizobacteria strains on root-knot nematode, *Meloidogyne incognita*, on tomatoes. Egypt. J. Biol. Pest Control.

[23] Hu Y., You J., Wang Y., Long Y., Wang S., Pan F., Yu Z. (2022). Biocontrol efficacy of *Bacillus velezensis* strain YS-AT-DS1 against the root-knot nematode *Meloidogyne incognita* in tomato plants. Front. Microbiol..

[24] Gao H., Qi G., Yin R., Zhang H., Li C., Zhao X. (2016). *Bacillus cereus* strain S2 shows high nematicidal activity against *Meloidogyne incognita* by producing sphingosine. Sci. Rep..

[25] Du J., Gao Q., Ji C., Song X., Liu Y., Li H., Li C., Zhang P., Li J., Liu X. (2022). *Bacillus licheniformis* JF-22 to control *Meloidogyne incognita* and its effect on tomato rhizosphere microbial community. Front. Microbiol..

[26] Bel Y., Galeano M., Baños-Salmeron M., Andrés-Antón M., Escriche B. (2024). *Bacillus thuringiensis* Cry5, Cry21, App6 and Xpp55 proteins to control *Meloidogyne javanica* and *M. incognita*. Appl. Microbiol. Biotechnol..

[27] Yao Y., Wang L., Zhai H., Dong H., Wang J., Zhao Z., Xu Y. (2025). *Bacillus velezensis* A-27 as a potential biocontrol agent against *Meloidogyne incognita* and effects on rhizosphere communities of celery in field. Sci. Rep..

[28] Zhang R., Ouyang J., Xu X., Li J., Rehman M., Deng G., Shu J., Zhao D., Chen S., Sayyed R.Z. (2022). Nematicidal activity of *Burkholderia arboris* J211 against *Meloidogyne incognita* on tobacco. Front. Microbiol..

[29] Kim J.-H., Lee B.-M., Kang M.-K., Park D.-J., Choi I.-S., Park H.-Y., Lim C.-H., Son K.-H. (2023). Assessment of nematicidal and plant growth-promoting effects of *Burkholderia* sp. JB-2 in root-knot nematode-infested soil. Front. Plant Sci..

[30] Stucky T., Hochstrasser M., Meyer S., Segessemann T., Ruthes A.C., Ahrens C.H., Pelludat C., Dahlin P. (2023). A novel robust screening assay identifies *Pseudomonas* strains as reliable antagonists of the root-knot nematode *Meloidogyne incognita*. Microorganisms.

[31] Abd El-Aal E.M., Shahen M., Sayed S., Kesba H., Ansari M.J., El-Ashry R.M., Aioub A.A.A., Salma A.S.A., Eldeeb A.M. (2021). In vivo and in vitro management of *Meloidogyne incognita* (Tylenchida: Heteroderidae) using rhizosphere bacteria, *Pseudomonas* spp. and *Serratia* spp. compared with oxamyl. Saudi J. Biol. Sci..

[32] Burkett M., Kokalis-Burelle N., Lawrence K., van Santen E., Kloepper J. (2008). Suppressiveness of root-knot nematodes mediated by rhizobacteria. Biol. Control.

[33] El-Hadad M.E., Mustafa M.I., Selim S.M., El-Tayeb T.S., Mahgoob A.E.A., Aziz N.H.A. (2011). The nematicidal effect of some bacterial biofertilizers on *Meloidogyne incognita* in sandy soil. Braz. J. Microbiol..

[34] De Nardo E.A.B., Grewal P.S. (2003). Compatibility of *Steinernema feltiae* (Nematoda: *Steinernematidae*) with pesticides and plant growth regulators used in glasshouse plant production. Biocontrol Sci. Technol..

[35] Shi Q.Q., Zhang J., Fu Q., Hao G.Y., Liang C., Duan F.M., Zhao H.G., Song W.W. (2024). Biocontrol efficacy and induced resistance of *Paenibacillus polymyxa* J2-4 against *Meloidogyne incognita* infection in cucumber. Biol. Control Microb. Ecol..

[36] Buchanan R.E., Gibbons N.E. (1984). Bergey’s Manual of Determinative Bacteriology.

[37] Zhao D., Zhao H., Zhao D., Zhu X.F., Wang Y.Y., Duan Y.X., Xuan Y.X., Chen L.J. (2018). Isolation and identification of bacteria from rhizosphere soil and their effect on plant growth promotion and root-knot nematode disease. Biol. Control.

[38] Ei S.L., Lwin K.M., Padamyar, Khaing H.O., Yu S.S. (2018). Study on IAA Producing Rhizobacterial Isolates and Their Effect in Talc-Based Carrier on Some Plants. J. Soil Sci. Plant Health.

[39] Tang Y.W., Bonner J. (1948). The enzymatic inactivation of indole acetic acid. II. The physiology of the enzyme. Am. J. Bot..

[40] Gordon S.A., Weber R.P. (1951). Colorimetric estimation of inodole3-acetic acid. Plant Physiol..

[41] Zhang T., Wang X.L., Zhou J., Zhou W., Zhou S.Q. (2025). Construction of phosphate-solubilizing microbial consortium and its effect on the remediation of saline-alkali soil. Microb. Ecol..

[42] Hussey R.S., Barker K.R. (1973). A comparison of methods of collecting inocula of *Meloidogyne* spp. Including a new technique. Plant Dis. Rep..

[43] Bridge J., Page S.L. (1980). Estimation of root-knot nematode infestation levels on roots using a rating chart. Int. J. Pest Manag..

[44] Ayaz M., Ali Q., Farzand A., Khan A.R., Ling H., Gao X. (2021). Nematicidal volatiles from *Bacillus atrophaeus* GBSC56 promote growth and stimulate induced systemic resistance in tomato against *Meloidogyne incognita*. Int. J. Mol. Sci..

[45] Saharan R., Patil J.A., Yadav S., Kumar A., Goyal V. (2023). The nematicidal potential of novel fungus, *Trichoderma asperellum* FbMi6 against Meloidogyne incognita. Sci. Rep..

[46] Sharma I.P., Sharma A.K. (2017). Effective control of root-knot nematode disease with *Pseudomonad* rhizobacteria filtrate. Rhizosphere.

[47] Hussain T., Haris M., Shakeel A., Ahmad G., Khan G.A., Khan M.A. (2020). Bio-nematicidal activities by culture filtrate of *Bacillus subtilis* HussainT-AMU: New promising biosurfactant bioagent for the management of Root Galling caused by *Meloidogyne incognita*. Vegetos.

[48] Zhai Y., Shao Z., Cai M., Zheng L., Li G., Huang D., Cheng W., Thomashow L.S., Weller D.M., Yu Z. (2018). Multiple modes of nematode control by volatiles of *Pseudomonas putida* 1A00316 from Antarctic soil against *Meloidogyne incognita*. Front. Microbiol..

[49] Rao M.S., Kamalnath M., Umamaheswari R., Rajinikanth R., Prabu P., Priti K., Grace G.N., Chaya M.K., Gopalakrishnan C. (2017). *Bacillus subtilis* IIHR BS-2 enriched vermicompost controls root knot nematode and soft rot disease complex in carrot. Sci. Hortic..

[50] Yin N., Zhao J.L., Liu R., Li Y., Ling J., Yang Y.H., Xie B.Y., Mao Z.C. (2021). Biocontrol efficacy of *Bacillus cereus* strain Bc-cm103 against *Meloidogyne incognita*. Plant Dis..

[51] Islam S., Akanda A.M., Prova A., Islam M.T., Hossain M.M. (2016). Isolation and identification of plant growth promoting rhizobacteria from cucumber rhizosphere and their effect on plant growth promotion and disease suppression. Front. Microbiol..

[52] Khamsuk K., Dell B., Pathom-Aree W., Pathaichindachote W., Suphrom N., Nakaew N., Jumpathong J. (2024). Screening plant growth-promoting bacteria with antimicrobial properties for upland rice. J. Microbiol. Biotechnol..

[53] Ghorbel S., Aldilami M., Zouari-Mechichi H., Mechichi T., AlSherif E.A. (2023). Isolation and characterization of a plant growth-promoting rhizobacterium strain MD36 that promotes barley seedlings and growth under heavy metals stress. 3 Biotech.

[54] Lau E.T., Tani A., Khew C.Y., Chua Y.Q., Hwang S.S. (2020). Plant growth-promoting bacteria as potential bio-inoculants and biocontrol agents to promote black pepper plant cultivation. Microbiol. Res..

[55] Dolu H., Killi D., Bas S., Bilecen D.S., Seymen M. (2025). Effectiveness of salt priming and plant growth-promoting bacteria in mitigating salt-induced photosynthetic damage in melon. Photosynth. Res..

[56] Spaepen S., Bossuyt S., Engelen K., Marchal K., Vanderleyden J. (2014). Phenotypical and molecular responses of *Arabidopsis thaliana* roots as a result of inoculation with the auxin-producing bacterium *Azospirillum brasilense*. New Phytol..

[57] Finkel O.M., Salas-González I., Castrillo G., Conway J.M., Law T.F., Teixeira P.J.P.L., Wilson E.D., Fitzpatrick C.R., Jones C.D., Dangl J.L. (2020). A single bacterial genus maintains root growth in a complex microbiome. Nature.

[58] Turan M., Gulluce M., Von Wirén N., Sahin F. (2012). Yield promotion and phosphorus solubilization by plant growth–promoting rhizobacteria in extensive wheat production in Turkey. J. Plant Nutr. Soil Sci..

[59] Marschner P., Crowley D., Rengel Z. (2011). Rhizosphere interactions between microorganisms and plants govern iron and phosphorus acquisition along the root axis—Model and research methods. Soil Biol. Biochem..

[60] Iniguez A.L., Dong Y., Triplett E.W. (2004). Nitrogen fixation in wheat provided by *Klebsiella pneumoniae* 342. Mol. Plant Microbe Interact..

[61] Singh T.B., Sahai V., Goyal D., Prasad M., Yadav A., Shrivastav P., Ali A., Dantu P.K. (2020). Identification, characterization and evaluation of multifaceted traits of plant growth promoting rhizobacteria from soil for sustainable approach to agriculture. Curr. Microbiol..

[62] Syed Ab Rahman S.F., Singh E., Pieterse C.M.J., Schenk P.M. (2018). Emerging microbial biocontrol strategies for plant pathogens. Plant Sci..

[63] Ye L., Wang J.-Y., Liu X.-F., Guan Q., Dou N.-X., Li J., Zhang Q., Gao Y.-M., Wang M., Li J.-S. (2022). Nematicidal activity of volatile organic compounds produced by *Bacillus altitudinis* AMCC 1040 against *Meloidogyne incognita*. Arch. Microbiol..

