# Supplementary figures and images for: Isolation and Characterization of Pseudomonas aeruginosa XR2-39 Against Meloidogyne incognita and Its Enhancement of Tomato Growth
Source: Microorganisms. 2025 Dec 19;14(1):5. doi: 10.3390/microorganisms14010005 (PMC12844080; doi:10.3390/microorganisms14010005)

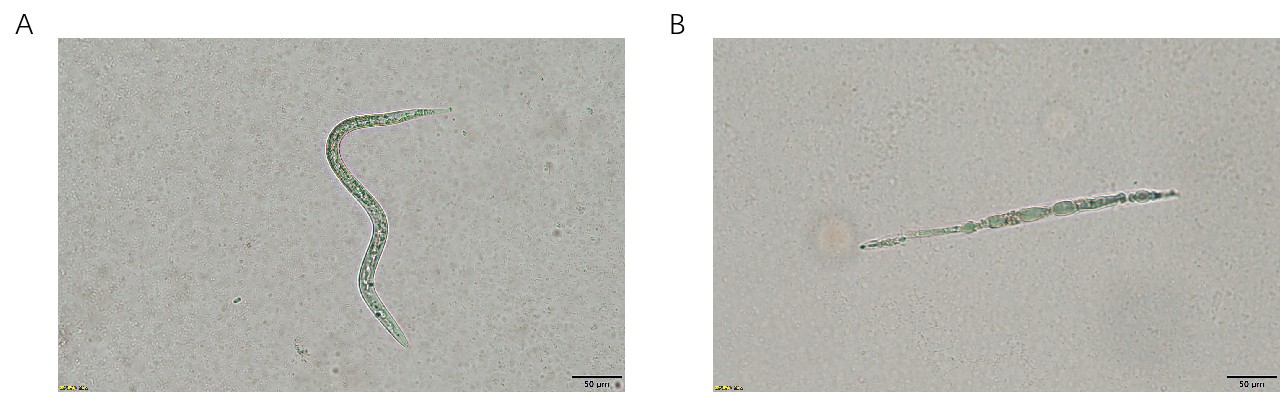

Supplement: Supplementary file 1 [file microorganisms-14-00005-s001.zip › microorganisms-4034052-supplementary.jpg]
